# Trauma-involved care: knowledge, attitudes, and practices of mental health professionals

**DOI:** 10.1192/j.eurpsy.2025.904

**Published:** 2025-08-26

**Authors:** F. İnci, F. Oflaz, A. Büyükbayram Arslan, B. Şimşek Arslan, N. Kılıç

**Affiliations:** 1 Health Science Faculty, Niğde Ömer Halis Demir University, Nevşehir; 2 School of Nursing, Koç University, İstanbul; 3 Health Science Faculty, İzmir Katip Çelebi University, İzmir; 4 Health Science Faculty, Burdur Mehmet Akif ErsoyUniversity, Burdur; 5 Health Science Faculty, Artvin Çoruh University, Artvin, Türkiye

## Abstract

**Introduction:**

The number of people exposed to trauma has increased due to disasters, epidemics, wars, and various man-made causes occurring worldwide- including Turkey. Therefore, the principles of trauma-informed care should be applied in all healthcare settings and incorporated into policies and institutional frameworks (Saunders et al., 2023; Greer, 2023; Huo et al., 2023). Although mental health professionals are experienced in the effects of trauma, a more systematic approach is required to enhance awareness of and achieve widespread trauma-informed care. In Turkey, there is no standardized guide or algorithm for providing care and treatment to trauma victims, and no guide has been developed to ensure a common understanding of care in approaching trauma among mental health professionals.

**Objectives:**

This study examined the knowledge, skills, and stance of professionals providing mental health services in Turkey regarding trauma-informed care, as well as their intervention skills related to trauma. This constitutes the first phase of the studies to establish a fundamentally trauma-informed care model in existing mental health settings.

**Methods:**

The study was conducted with a cross-sectional design, and the convenience sampling method was used. The study population was mental health services professionals in Turkey. The participants were recruited by researchers through social media platforms, and only those accepting participation were included in the study. The study was completed with 197 participants who filled out the survey forms.The data were collected using the Trauma-Informed Care Scale and the Trauma Intervention Skills Scale through an online survey. The data were analyzed using Spearman correlation analysis, Mann Whitney U, and Kruskal Wallis tests.

**Results:**

More than half of the participants were unaware of the concept of trauma-informed care (55.3%), and only 14.2% felt competent in presenting this care model. In trauma-informed care scores, gender, age, and education level did not create a significant difference (p>0.05), while those who have received information about trauma-informed care during their years of education scored higher (p<0.05). Compared to other professionals, nurses had higher trauma-informed care scores, and psychologists had an augmented ability to intervene in trauma patients.

**Conclusions:**

The results of the research indicated that while mental health professionals possess knowledge about trauma, they did not reach the desired level in developing attitudes towards trauma-informed care and feeling competent in this area. Trauma-informed care seemed more about providing information related to procedures performed in the clinic or ensuring that the patient was not traumatized during the procedure. Therefore, more studies are needed to evaluate TIC practices in the much-needed mental health field.

**Disclosure of Interest:**

None Declared

